# On atom–atom ‘short contact’ bonding interactions in crystals^1^

**DOI:** 10.1107/S2052252515002067

**Published:** 2015-02-26

**Authors:** Claude Lecomte, Enrique Espinosa, Cherif F. Matta

**Affiliations:** a Laboratoire de Cristallographie, Résonance Magnétique et Modélisations, UMR CNRS 7036, Institut Jean Barriol, Faculté des Sciences et Technologies, Université de Lorraine, BP 70239, Boulevard des Aiguillettes 54506 Vandoeuvre-lès-Nancy France; b Department of Chemistry and Physics, Mount Saint Vincent University, Halifax, Nova Scotia, B3M 2J6 Canada; c Department of Chemistry, Dalhousie University, Halifax, Nova Scotia, B3H 4J3, Canada; d Department of Chemistry, Saint Mary’s University, Halifax, Nova Scotia, B3H 3C3, Canada

**Keywords:** bond paths in crystals, crystal binding, crystal stability, electron density, quantum theory of atoms in molecules (QTAIM), high-resolution X-ray diffraction, synthons

## Abstract

Professor Dunitz questions the usefulness of ascribing crystalline structural stability to individual atom–atom intermolecular interactions viewed as bonding (hence stabilizing) whenever linked by a bond path. An alternative view is expressed in the present essay that articulates the validity and usefulness of the bond path concept in a crystallographic and crystal engineering context.

## Abstract 2

The electron density distribution *ρ*(**r**) contains the information needed to quantitatively analyze bonding interactions between atoms, exhibiting short, medium or long inter-nuclear distances. Atom–atom bonding interactions, the orientation of molecules in the space and crystal structure are all inter-determined.

Professor Jack D. Dunitz has questioned the meaning of interatomic short contacts in intermolecular regions of crystals and whether they should be regarded as bonding interactions (Dunitz, 2015 ▶). Professor Dunitz concludes his thought-provoking essay with the question: ‘*should the observation of short distances between pairs of atoms on the peripheries of different molecules in crystals be regarded as evidence of specific intermolecular bonding between the atoms concerned*?’, then adds the suggestion that ‘*if the answer is not yes but no or perhaps or sometimes: how are we to distinguish the bonding atom–atom interaction from the energetically neutral or anti-bonding type*? *Do we need another IUPAC commission to decide*?’ It is the purpose of the present essay to examine critically some of the statements made and to express an alternative viewpoint.

To start, the entire essay of Professor Dunitz is centered on the idea that one could assign different positions to distinct atom pairs on their respective atom–atom interaction potentials within a crystal. Any molecule or crystal system is univocally defined by its atoms and the relative positions of their nuclei in space. This atomic description, which is commonly called the molecular structure, is determined by the associated electron density distribution *ρ*(**r**) of the system. There exists a mapping between the molecular structure and *ρ*(**r**) observed from a diffraction experiment or calculated from electronic structure theory. The credit for discovering that mapping goes to the late Richard F. W. Bader (1931–2012) in the form of his now-used Quantum Theory of Atoms in Molecules (QTAIM) (Bader, 1990 ▶). From a given nuclear arrangement (molecular geometry), the electron density *ρ*(**r**) uniquely determines the forces that act on the nuclei, which are classical electrostatic forces as stated by the Hellmann–Feynman theorem (Feynman, 1939 ▶; Hellmann, 1937 ▶). In turn, the total energy of the system is a functional of *ρ*(**r**) *via* the Hohenberg–Kohn (HK) theorem (Hohenberg, 1964 ▶) of the Density Functional Theory (DFT) (Hohenberg, 1964 ▶; Parr & Yang, 1989 ▶). The HK theorem is in fact much more far reaching since it states that *ρ*(**r**), by uniquely specifying the external potential, fixes the Hamiltonian and hence all ground and excited properties since it determines the eigenstates themselves. From this picture, the *ρ*(**r**) function governs all interactions in the system and emerges as the conceptual bridge between structure and properties of a system, which can be made by one, a small cluster, or a quasi-infinite number of molecules or ions as in a crystal.

A crystal is a time-averaged equilibrium configuration of atoms in space where nuclei are vibrating around equilibrium positions. A crystal where any atomic nucleus experiences a net repulsion or attraction from its crystallographic surroundings is yet to exist, whether in the real physical world or in a computer model of this world. The reason is simple: any displacement from equilibrium geometry, where Hellman–Feynman forces (Feynman, 1939 ▶; Hellmann, 1937 ▶) on all nuclei in the crystal vanish, will create a restoring force bringing all nuclei back to their equilibrium positions. What then is the meaning of an attractive or repulsive interaction in an existing crystal where every atom vibrates around its equilibrium position at the bottom of a global multidimensional potential well? It should be clear that attributing a ‘bonded interaction’ to a net ‘attraction’ and a ‘non-bonded’ or ‘close contact’ to a net ‘repulsion’ is not only misleading and incorrect, but is in fact meaningless because, on average, there are no net forces on any nucleus (Hellman-Feynman force) or atom (Ehrenfest force) in a crystal.

Observing bond paths and their associated (3,−1) bond critical points (BCPs) is sufficient to establish a bonding interaction (Bader, 1990 ▶; Bader, 1998 ▶; Runtz *et al.*, 1977 ▶), whether intra- or inter-molecular (covalent, ionic, metal–metal, metal–ligand, hydrogen, halogen, chalcogen, van der Waals, hydrogen⋯hydrogen (H⋯H) bonding, *etc*.) When bond paths are observed where classical models prohibit bonding, these cases may indicate a failure of these models to encapsulate unusual bonding for which they were never designed. Often, discrepancies between classical models and what the observable topology and topography of *ρ*(**r**) is telling us turn out to be cases of great interest. An example is the concept of H⋯H bonding (Cukrowski & Matta, 2010 ▶; Hernández-Trujillo & Matta, 2007 ▶; Matta *et al.*, 2003 ▶), which after a wave of initial criticism, as it violates classical models, has growingly proved to be useful, predictive, and consistent with observation (Paul *et al.*, 2011 ▶; Echeverría *et al.*, 2011 ▶; Monteiro & Firme, 2014 ▶; Sabirov, 2014 ▶). In the case of challenging experimental charge density determinations, such as those describing halogen and chalcogen heavy atoms involved in very weak interactions, the experimental properties of the topology of *ρ*(**r**) in the intermolecular regions are systematically supported by theoretical calculations and *vice versa*. As an example, joint experimental and periodic theoretical charge density studies of Br- and Se-compounds involving very weak interactions (Brezgunova *et al.*, 2012 ▶, 2013 ▶) indicate reproducible bond paths and *ρ*(**r**) features in the intermolecular regions, as well as in the close vicinity of the heavy atoms.

Dunitz endorses a conjecture in a comment (Spackman, 1999 ▶) on our paper on hydrogen bond (HB) energetics from topological analysis of experimental *ρ*(**r**) (Espinosa *et al.*, 1998 ▶) that the overlap of spherical electron densities *ρ* ^IAM^(**r**) (promolecular densities derived from an independent atom model, IAM) reproduces bond paths similar to those obtained experimentally or from a fully fledged quantum-mechanical calculation. Dunitz’s statement can be rephrased as a question: can we use descriptors derived from IAM where, by construction, the system is not at equilibrium because the electron configuration is artificial, in the same manner as we use descriptors derived from the real *ρ*(**r**)? Even in those cases where the topological properties of *ρ* ^IAM^(**r**) may be close to the experimental crystal density *ρ* ^crystal^ **(r**), systematic deviations from the latter clearly indicate that IAM yields unreliable non-physical densities. This is observed, for instance, in the case of hydrogen-bonding interactions. Indeed, in the ∇^2^ *ρ*_BCP_ *versus* ρ_BCP_ plot of the H⋯O interactions analyzed in Spackman’s paper, the comparison of *ρ* ^IAM^(**r**) with *ρ* ^crystal^ **(r**) shows a systematic deviation of *ρ* ^IAM^(**r**) with respect to *ρ* ^crystal^ **(r**), except for a few systems discussed in the article. As pointed out by the author, ‘*experimental electron densities systematically yield values of the Laplacian at the bond critical point considerably greater than predicted by the promolecule for the same value of ρ*_BCP_’ (Spackman, 1999 ▶). These results indicate that *ρ* ^IAM^(**r**) is systematically less depleted than expected. Unsurprisingly, the artificial IAM fails to describe the correct behavior of closed-shell interactions, where electrons in closed electronic shells exclude those of same spins of another nearby closed shell, leading to depletion of *ρ* **(r**) and a strongly positive ∇^2^ *ρ* at intermolecular BCPs. Consequently, if one seeks more than just an approximate set of nuclear coordinates (geometrical structure), as obtained in routine X-ray determinations for which a promolecule model may be sufficient, additional *crucial* information must be determined from the experiment if a physically meaningful description of hydrogen bonding is required. Other studies involving C–H⋯O hydrogen bonding interactions reached a similar conclusion (Gatti *et al.*, 2002 ▶), that promolecular densities can differ from experimental determination or theoretical calculation of *ρ* **(r**) in significant ways with regards to electron densities at BCPs and even, in some cases, the topology of the density itself.

Further, claims that promolecular densities are essentially sufficient for a topological analysis ignore the subtleties of bonding that the electron density topology captures, for example in borderline cases such as fluxional bonding and (C=C) π-metal bonding (Macchi *et al.*, 1998 ▶). These claims are also not aligned with the observation that physical proximity *alone* is not a sufficient condition for the presence of a bond path in a crowded system nor does it automatically prepare the system for an incipient bonding interaction reflected, for example, in relatively high delocalization indices. This last observation is exemplified by a crowded titanium metalorganic (Tomaszewski *et al.*, 1998 ▶) in which two C atoms placed at 2.338 (6) and 2.299 (6) Å from the central Ti atom are linked to it by a bond path, whereas no bond path is found for another C atom lying at an equivalent short distance [2.293 (7) Å]. The delocalization indices are also not falling monotonically with distance, being 0.11 and 0.14*e* ^−^, respectively, shared between the first two C atoms and the Ti atom, and only 0.06*e* ^−^ for the third C atom (Bader & Matta, 2001 ▶). There is thus much more to the bond path and other QTAIM indicators of bonding than mere physical proximity, which is the only effect captured in a promolecular density.

Another central and final question raised in Professor Dunitz’s essay is: do individual atom–atom pair interactions determine the crystal structure or is the packing of the molecules in the crystal a consequence of the whole interactions between molecular charge distributions? Crystal engineering tools are based on synthons (Corey, 1967 ▶; Desiraju, 1995 ▶). Synthons operate as a consequence of molecular recognition of interacting functional groups, a recognition that has been given a physical basis in QTAIM as a complementarity of regions of electronic charge concentration (Lewis base-like regions) with interacting regions of charge depletion (Lewis acid-like regions) (Bader, 1990 ▶). In this theory, it has been recognized that the three-dimensional second derivative of the total electron density, that is its Laplacian [∇^2^ *ρ*(**r**)], is the scalar field that embodies this acid–base complementarity (Bader, 1990 ▶) and hence governs the interaction patterns of synthons. Recent experimental studies using the topological analysis of the Laplacian of the electron density demonstrate that the orientation of atom–atom interactions (and therefore that of the corresponding intermolecular interactions) is indeed governed by electrophilic/nucleophilic interactions of regions of charge concentration (CC) with regions of charge depletion (CD) in the atomic valence shells of the atom–atom pair interactions (Bui *et al.*, 2009 ▶). This complementarity, which has also been investigated in halogen, chalcogen and weak hydrogen bonding (Brezgunova *et al.*, 2012 ▶, 2013 ▶), is such that the CC–CD directions are almost completely aligned with the given internuclear atom–atom directions, even for interactions with energies estimated as low as <5 kJ mol^−1^. Bond paths and CC–CD interactions are clearly indicative of atom–atom interactions.

It is important to emphasize that hydrogen bonding (HB) cannot be invoked as a somewhat unique type of bonding interaction holding a molecular crystal together, since synthons can involve intermolecular interactions other than HBs. Why should HB be an exception and described separately from other interactions contributing to the crystalline stability? Aren’t the wide range of HB energies (which brackets weak van der Waals up to covalent interactions) and the variability of donors and acceptors [neutral, positively, negatively (fully/partially) charged] indicative of a versatility of HB that blurs the artificially drawn boundaries distinguishing it from other bonding intermolecular interactions? Individual atom–atom pair interactions (of atoms in a crystal) *are* structure determining. Indeed, as recently observed in molecular crystals of 4-nitroimidazole derivatives (Poulain *et al.*, 2014 ▶), a contamination of 2% Br in the 5-position (C–Br) replacing 5-carbonitrile (C–CN) changes the crystal structure completely: in the pure crystal, the main intermolecular interactions are halogen bonding CN⋯Cl–C, while in the crystal of the 2% solid solution dipolar antiparallel CN⋯CN are favored; these interactions are fully characterized by their bond paths and critical points. The 2% solid solution crystal originates from antiparallel C–Br⋯Br–C or C–Br⋯CN–C synthons which drive the orientation of the molecules and therefore the new crystal packing.
